# A new paradigm for epidermal growth factor receptor expression exists in PTC and NIFTP regulated by microRNAs

**DOI:** 10.3389/fonc.2023.1080008

**Published:** 2023-04-11

**Authors:** Abeer Al-Abdallah, Iman Jahanbani, Rola H. Ali, Nabeel Al-Brahim, Jeena Prasanth, Bashayer Al-Shammary, Maie Al-Bader

**Affiliations:** ^1^ Pathology Department, Kuwait University, Faculty of Medicine, Kuwait City, Kuwait; ^2^ Pathology Department, Farwaniya Hospital, Kuwait City, Kuwait; ^3^ Physiology Department, Kuwait University, Faculty of Medicine, Kuwait City, Kuwait

**Keywords:** papillary thyroid carcinoma, PTC, NIFTP, miRNA, EGFR

## Abstract

**Intoduction:**

Identification of molecular alterations associated with tumor behavior is necessary to guide clinical management. The 2022 WHO classification has organized the thyroid follicular cell-derived neoplasms into benign, low-risk and high-risk neoplasms, and emphasized the value of biomarkers that may provide differential diagnostic and prognostic information to avoid overtreatment of low risk neoplasms. This work aims to study the epidermal growth factor receptor (EGFR) expression, functional and spatial dynamics in relation to specific miRNAs alterations in papillary thyroid cancer (PTC) and in non-invasive follicular thyroid neoplasm with papillary-like nuclear features (NIFTP) considered as models of high-risk and low-risk thyroid tumors respectively.

**Methods:**

Primary thyroid cultured cells were used for miRNA gain/loss of function and luciferase reporter assays. Paraffin embedded tissues were used for real time PCR, immuno-fluorescence stain and confocal microscopy experiments.

**Results:**

Our results showed that in PTC, EGFR mRNA is reduced as an effect of miR-146b-5p upregulation. The EGF expression is low and the ERK pathway is inhibited. The EGFR protein high cytoplasmic expression and colocalization with the endosomal/exosomal markers, ALIX and CD63, suggest the occurrence of stress-induced EGFR internalization, accumulation in endosomal vesicles and secretion *via* exosomes. In NIFTP EGFR transcription is increased in association with downregulation of miR-7-5p and the EGFR/ERK pathway is active indicating dependence on the canonical EGFR pathway for growth.

**Conclusion:**

Downregulation of transcript level along with cytoplasmic accumulation of undegraded protein is a new pattern of EGFR regulation associated with malignancy in thyroid. Further research is needed to elucidate the intracellular trafficking defects responsible for this specific EGFR dynamic in PTC.

## 1 Introduction

The epidermal growth factor receptor (EGFR) is the prototype of tyrosine kinase receptors which contributes to human cancer development and progression (1, 2). Altered EGFR signaling is considered one of the most dysregulated molecular pathways caused either by EGFR gene mutation or amplification, overexpression due to dysregulated transcription and/or translation, or other alterations of the normal regulatory mechanisms (3–5). EGFR overexpression has been reported in many types of cancer and has been associated with aggressive behavior and poor prognosis (6–9). Its well-established role in promoting cell proliferation and survival has led to the discovery and application of many therapeutic drugs targeting the EGFR pathway (10, 11). However, targeted EGFR therapy has shown limited clinical success in cancer patients (12–15). Extensive research into the reasons of drug resistance highlighted the high complexity of EGFR signaling and regulation during cancer cell progression and interaction with the microenvironment. EGFR performs different functions depending on the dynamics of its intracellular trafficking and subcellular location and in response to different stimuli (16–18). Altered receptor endocytosis and trafficking and cellular stresses activate the wild-type EGFR signaling thus diminishing the dependence on gene mutation (19). Moreover, EGFR performs functions that are independent of its tyrosine kinase activity in cancer cells which necessitate new perspectives in assessing EGFR for cancer management (20, 21).

The incidence of thyroid cancer has been increasing worldwide, largely driven by the increase in papillary thyroid cancer (PTC) (22–24). In the new WHO classification, Thyroid tumors are divided into benign, low-risk, and malignant neoplasms with deeper consideration of the cell of origin, pathologic features, molecular classification, and biological behavior (25). The multifocal hyperplastic/neoplastic lesions used to be called “multinodular goiter” is now referred to as “thyroid follicular nodular disease (FND)”. The low-risk follicular cell–derived neoplasms included the non-invasive follicular thyroid neoplasm with papillary-like nuclear features (NIFTP) among other lesions. The malignant follicular cell–derived neoplasms were stratified based on molecular profiles and aggressiveness and included classic PTC (PTC), as a high-risk subtype, among other types (25). PTC represent the BRAF-like malignancies, associated with altered regulation of the mitogen-activated protein kinase (MAPK) and stimulation of the extracellular signal–regulated kinases (ERK) transcriptional programs that mediate tumorigenesis and progression to malignancy (26, 27). Although multiple genes and cellular pathways have been reported to contribute to PTC pathogenesis, there is currently no conclusive data on their functions or clinical utility (28–30). EGFR was reported to be overexpressed in anaplastic thyroid carcinomas, follicular thyroid carcinomas and in primary medullary carcinomas, with no evidence of somatic EGFR mutations (31–34). EGFR tyrosine kinase inhibitor is undergoing phase II testing for the treatment of patients with iodine-refractory advanced thyroid carcinoma with no significant clinical benefit yet available (35, 36). In PTC, wild type EGFR overexpression was reported as an important biomarker of aggressive behavior (37–39). However, other studies reported no association of EGFR overexpression with adverse clinical features in PTC (40).

miRNAs are promising biomarkers in the diagnosis, prognosis and therapy of cancer including PTC (41–43). In our previous work, we showed that downregulation of miR-7-5p significantly discriminates thyroid neoplasms including classic PTC (PTC), follicular variant PTC and NIFTP from hyperplastic thyroid lesions (now called FND). We also showed that miR-146b-5p high expression is characteristic of PTC, is upregulated in the circulation in preoperative blood samples and is significantly reduced post thyroidectomy (44). Functional studies, including ours, suggested that miRNA-146b-5p is involved in the pathogenesis of PTC (42, 45, 46). Interestingly, both miR-7-5p and miR-146b-5p can target EGFR by binding to the 3′UTR region of its mRNA. EGFR has been reported as one of the targets of miR-7-5p in breast, ovarian and lung cancers and in glioblastoma (47–50). Mechanistic evidence showed that miR-7-5p can inhibit the proliferation of cancer cells through regulating the expression of EGFR (51). miR-146b-5p was found to suppress the expression of EGFR in human glioblastoma cell lines (52). miR-146b-5p was suggested as a useful tool for overcoming EGFR resistance through regulation of miR-146b-5p/IRAK1/NF-κB signaling (53). miR-146b-5p overexpression was suggested as a new tool to improve the clinical benefit of EGFR-targeted treatments in ovarian cancer patients (54). There are no reports on the interaction of miR-7-5p and miR-146b-5p with EGFR in PTC or on the expression of EGFR in the newly classified tumors such as NIFTP. In this work, we are investigating the expression of EGFR and its regulation by miR-7-5p and miR-146b-5p in PTC and NIFTP as models of high risk and low risk thyroid tumors respectively.

## 2 Materials and methods

### 2.1 Patients

Thyroid tissue samples from partial or total thyroidectomy were obtained after completing routine gross and histopathological processing. Fresh tissue samples were used for culture (n=20). Formalin fixed paraffin embedded (FFPE) tissues were also obtained and microscopically reviewed by consultant histopathologists (R.H.A. and N.A.B.) following the new WHO classification of endocrine tumors (25). Samples included 50 classic papillary thyroid cancer (PTC) identified as the conventional infiltrative PTC composed predominantly of papillae, 20 noninvasive follicular neoplasm with PTC nuclear features (NIFTP), and 10 thyroid follicular nodular cases (FND). Ethical approval to conduct this study was obtained from Kuwait Ministry of Health and Kuwait University Health Sciences Center ethics committee.

### 2.2 Cell culture and transfection of miRNA inhibitor/mimic

Primary thyroid cell culture was done as described in our previous work (55). Cultured cells, at a density of 1 × 10^6^ cells, were transfected using 15 μL of HiPerFect Transfection Reagent (QIAGEN) and 150 ng of Anti has- miR-146b-5p miScript miRNA Inhibitor (QIAGEN) or Syn-hsa-miR-7-5p miScript miRNA Mimic (QIAGEN), or negative control siRNA (QIAGEN). In all transfection experiments endogenous miRNA levels were quantified by real-time PCR after transfection to ensure the success of the experiment. Oxidative stress was induced by treating the cancer cells with Paraquat (3 μM) for 24 h before analysis. This concentration of paraquat was optimized previously and proved to be the highest concentration that cause less than 10% cell death in thyroid cells culture (55).

### 2.3 Reverse transcription and real-time PCR amplification

Total RNA from cultured cells and from paraffin-embedded thyroid tissues was isolated using TRIzol (Ambion) and miRNeasy FFPE Kit (QIAGEN) respectively, following the manufacturers’ instructions. Genomic DNA elimination and reverse transcription of cDNA was done using the RT^2^ first strand Kit (QIAGEN) according to the manufacturer’s instructions. cDNA was mixed with RT^2^ SYBR Green Mastermix (QIAGEN), and RT^2^ qPCR Primer Assay specific primers (QIAGEN), and amplification reactions were set in 96-well plates. HPRT was used as housekeeping gene and PCR reactions were run on an ABI 7500 Fast Block real-time PCR machine. All samples were run in triplicates. Expression is calculated using the relative quantification method (ΔCt = CT of target normalized to CT of housekeeping genes). Expression in the test groups (Cells treated with miR146 inhibitor) are compared to control group (Cells treated with negative control) using the formula (ΔΔCT = ΔCT of test group - ΔCT of control group), and results are finally presented as fold change (2^–ΔΔCT^).

### 2.4 Luciferase reporter assay

The effect of miR-146b-5p and miR-7-5p on ERK and hypoxia signaling pathways was tested using the Cignal Finder Cancer Pathway Reporter Array (Qiagen) which uses the dual-luciferase technology. Firefly/Renilla activity ratios were generated for experimental (with miRNA inhibitor/mimic) and control transfections. The change in the activity of each signaling pathway is determined by comparing the normalized luciferase activities of the reporter in experimental versus control transfectants using the formula, Fold Change = (firefly/renilla ratio of experimental sample)/(firefly/renilla ratio of control sample). All transfections were performed in quadruplicate for each of the reporter assays. Transfection efficiency was estimated by observing GFP expression (a constitutively expressing GFP construct containing Monster Green^®^ Fluorescent Protein) in the positive control wells by fluorescence microscopy.

### 2.5 Immunofluorescence and immunohistochemistry stain

Expression and subcellular localization of EGFR, phosphorylated ERK (p42/44) and, phosphorylated p38 was tested by indirect immunofluorescence staining and confocal microscopy. Staining was done on FFPE tissue sections. The antibodies used were anti-EGFR (Cell Signaling, #42675), anti-phospho-ERK (Cell Signaling, RRID-AB-331775), anti-p38 (Cell Signaling, RRID-AB2139682), anti-ALIX (NOVUS, RRID-AB-960843) and, anti-CD63 (NOVUS, RRID-AB-108402). Primary antibodies were diluted according to manufacturers’ recommendations and incubated at 4°C overnight. Secondary antibodies labeled with Alexa Fluor 555 and Alexa Fluor 488 (Invitrogen), were incubated for one hour at room temperature. The nonspecific background was removed using blocking solution (DAKO). DAPI was used to counter stain the nuclei. Immuno-stained sections were visualized by laser scanning confocal microscope (LSM 700, Zeiss, Germany). Colocalization coefficients were calculated by Zen software (Zeiss, version 14.0.0.201, Germany) based on the co-occurrence of the red and green signals and their relative intensities. Colocalization coefficient of >0.5 was considered as positive co-expression.

### 2.6 *In Situ* Hybridization to study the expression of miR-146b-5p in thyroid tissues

Expression and cellular localization of miR-146b-5p was studied by ISH using miRCURY LNA miRNA Detection Probes (QIAGEN). The protocol parameters were first optimized with the LNA U6 snRNA positive control probe by adjusting the concentration of the probe, the hybridization temperature and proteinase K treatment. 100nM was selected as the optimal probe concentration and 55 degrees was selected as the optimal temperature for hybridization. Antigen retrieval was done using acetic acid pH=6 and heating for 10 min. Specific hybridization signals were detected as purple stain under light microscope. Combination of ISH procedure with immunofluorescence was done using miRNA probe labelled with FAM. Anti-FAM antibody was used as the primary antibody to detect the miRNA. Specific targets such as EGFR were detected using specific anti-EGFR antibody. Fluorescently labelled anti-sheep and anti-rabbit secondary Abs were used and the double immunofluorescence stain was examined by confocal microscopy.

### 2.7 Immunoblotting

Cultured and transfected thyroid cells was tested for change of expression of EGFR protein by Western immunoblot technique as described previously (55). 20 μg of protein was electrophoresed on SDS-PAGE gel and transferred to PVDF membranes at a stable current of 300 mA overnight at 4°C. The efficiency of transfer was confirmed by staining the gel with Coomassie blue stain. Blocking was done for 1 h at room temperature with 1x TBS with 1% Casein (Bio-Rad). Anti-EGFR antibody (Cell Signaling #42675) was incubated overnight at 4°C. Protein bands were detected by chemiluminescence using ECL-Plus kit (Amersham Pharmacia Biotech Ltd.). The density of the detected EGFR band was measured and compared to the cumulative densities of the total loaded proteins using ChemiDoc MP Imaging System (Bio-Rad) and Image Lab Software.

### 2.8 Statistical analysis

The expression level of genes in different groups were compared using student t-test. The difference in expression level between matched samples (treated with miRNA inhibitor/mimic versus control) was determined using paired-sample t-test using SPSS software. In all analysis, significance was considered with a p-value of <0.05.

## 3 Results

### 3.1 Expression of EGFR mRNA in thyroid clinical samples

Expression of EGFR mRNA was tested by RT-PCR in PTC, NIFTP and FND FFPE tissue samples. Results showed that EGFR expression is downregulated in PTC compared to NIFTP and FND, while it is upregulated in NIFTP compared to FND (**Table 1**). Moreover, miR-7-5p is downregulated in PTC and NIFTP compared to FND while miR-146b-5p is upregulated only in PTC (**Table 1**). No statistically significant association was detected between EGFR expression and aggressive pathological characteristics in PTC samples (**Table 2**). EGFR expression was found to be upregulated in samples with BRAF V600E mutation compared to BRAF mutation negative samples but with no statistical significance (**Table 2**).

**Table 1:** Expression of EGFR, miR-7-5p and miR-146b-5p in thyroid tissue samples.

	Mean expression value ΔCt (SD)			NIFTP vs. FND		PTC vs. FND		PTC vs. NIFTP	
	FND (n=10)	NIFTP (n=16)	PTC (n=28)	Fold Change	p-value*	Fold Change	p-value*	Fold Change	p-value*
**EGFR**	1.72 (1.09)	-2.82 (2.39)	3.04 (3.02)	23.35	<0.0001	-2.49	0.028	-58.10	<0.0001
**miR-7-5p**	-1.08 (1.16)	7.25 (6.07)	8.17 (5.17)	-321.80	0.0003	-608.87	<0.0001	1.89	0.082
**miR-146b-5p**	3.78 (1.06)	3.01 (2.87)	-0.68 (0.47)	1.71	0.788	22.03	<0.0001	12.92	0.0003

Expression is calculated using the relative quantification method (ΔCt = CT of target normalized to CT of housekeeping gene). Fold change is calculated using (2^–ΔΔCT^) formula (56). *Statistical analysis is done using student t-test.

**Table 2:** EGFR expression in relation to pathological characteristics in PTC samples.

Pathological characteristic							
		Invasion		Lymph node metastasis		BRAF mutation status	
	**Groups**	pos (n=26)	neg (n=14)	pos (n=14)	neg (n=26)	pos (n=16)	neg (n=25)
**EGFR**	**Average ΔCt**	2.81	3.17	2.56	3.20	1.86	3.80
	**Fold change (pos/neg)**	1.29		1.55		-3.84	
	p-value*	0.379		0.286		0.077	

Expression is calculated using the relative quantification method (ΔCt = CT of target normalized to CT of housekeeping gene). Fold change is calculated using (2^–ΔΔCT^) formula. *Statistical analysis is done using student t-test.

### 3.2 EGFR expression is regulated by miR-7-5p and miR-146b-5p in primary cultured thyroid cells

As miR-146b-5p is upregulated in PTC, we transfected cultured PTC cells with miR-146b-5p inhibitor to test the effect of miR-146b-5p downregulation on EGFR expression. Results showed that EGFR expression was significantly upregulated in cells transfected with miR-146b-5p inhibitor compared to control cells (**Table 3**). As miR-7-5p is downregulated in PTC, cultured PTC cells were transfected with miR-7-5p mimic. Transfection resulted in a significantly reduced expression of EGFR mRNA compared to control cells (**Table 4**).

**Table 3:** Effect of miR-146b-5p inhibitor on EGFR mRNA expression in PTC.

Samples	miR-146b-5p expression		EGFR Expression	
	Control	+/miR-146b-5p inhibitor	Fold Change	p-value*
**1**	-1.03	-1.41	-2.98	
**2**	73.01	-54.87	19.65	0.001
**3**	62.25	-134.13	3.02	
**4**	19.29	-176.27	10.75	
**5**	49.18	-28.60	39.25	
**6**	16.00	-4.50	22.57	
**7**	10.78	-2.91	140.40	

Fold change is calculated using the 2(-ΔΔCt) of the transfected versus control cells.

*Statistical analysis is done using paired student t-test.

**Table 4:** Effect of miR-7-5p mimic on EGFR mRNA expression in PTC.

Samples	miR-7-5p expression		EGFR Expression		
	Control	+/miR-7-5p mimic	Fold Change	p-value*	
**1**	-1.46	2.03	-20.32		
**2**	-90.51	3.92	-9.59	0.0009	
**3**	-63.12	4.35	-59.22		
**4**	-3.43	18.13	-3.90		
**5**	-2.97	3.25	-58.05		
**6**	-25.11	3.43	-142.22		

Fold change is calculated using the 2(-ΔΔCt) of the transfected cells versus control cells.

*Statistical analysis is done using paired student t-test.

### 3.3 Expression and subcellular localization of EGFR protein in thyroid tissues

**Figure 1:**
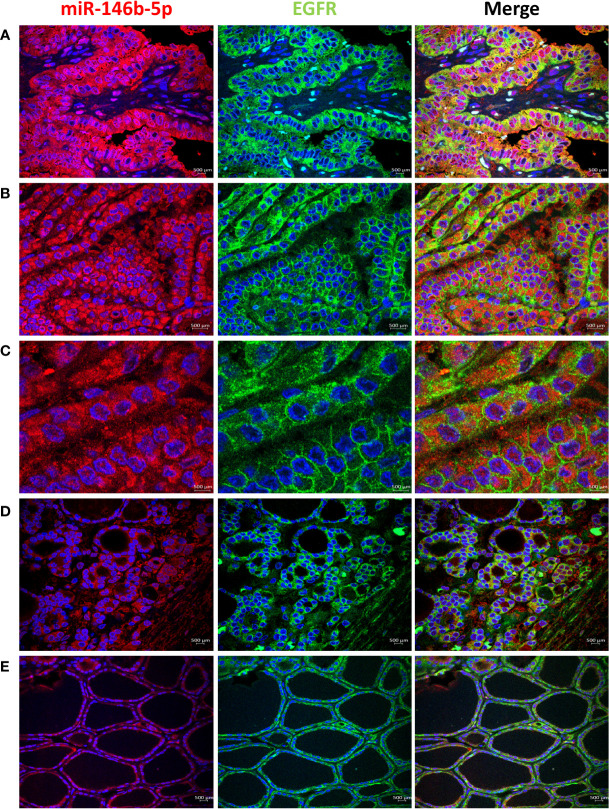
Expression of EGFR (green) and miR146b-5p (red) in representative samples. **(A)** PTC showing strong EGFR expression that colocalizes with miR-146 in the cytoplasm of tumor cells. **(B)** EGFR expression is enriched in the cell membrane in parts of the tissues in PTC. **(C)** In PTC cytoplasmic and membranous EGFR expression coexist in the same field and is not related to difference in miR-146b-5p level of expression. **(D)** NIFTP sample showing low cytoplasmic/membranous EGFR expression along with low miR-146b-5p expression in tumor cells. **(E)** FND sample showing low membranous EGFR expression and low miR-146b-5p in the follicular cells.

**Figure 2:**
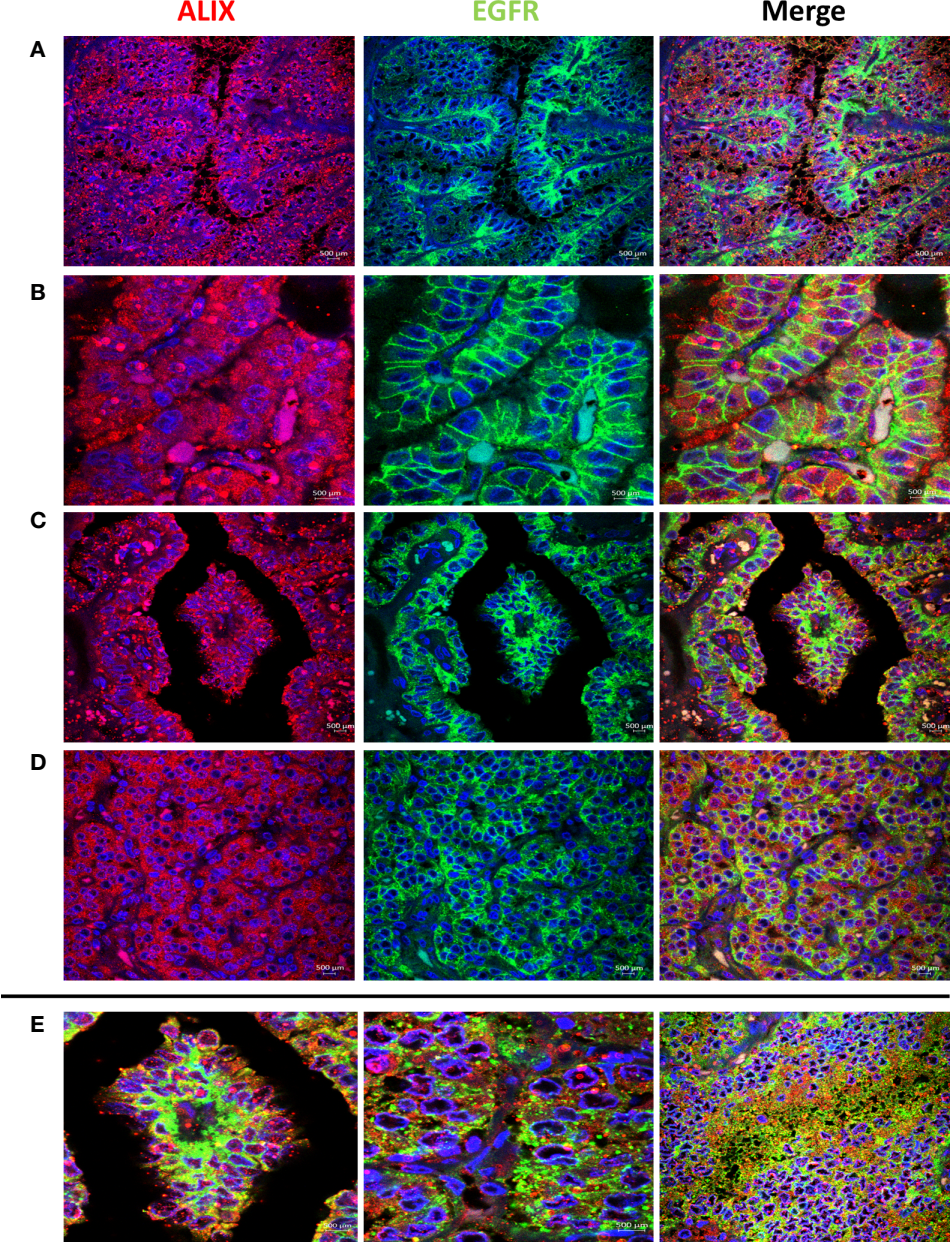
Representative samples stained with ALIX (red) and EGFR (green). Prominent ALIX positive vesicles of multiple sizes are seen in PTC in association with EGFR cytoplasmic expression **(A)**, EGFR membranous expression **(B)**, and EGFR enrichment at the basolateral surfaces **(C)**. **(D)** ALIX and EGFR expression in NIFTP sample. **(E)** Colocalization of ALIX and EGFR in intracellular and extracellular vesicles in PTC (Colocalization coefficient > 0.7).

**Figure 3:**
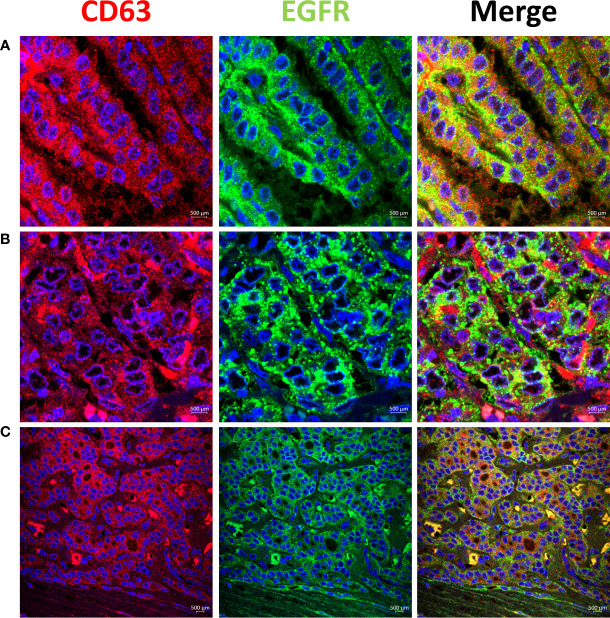
Representative samples stained with CD63 (red) and EGFR (green). CD63 colocalizes with EGFR in intracellular and extracellular vesicles in PTC (Colocalization coefficient >0.7) **(A, B)**. **(C)** In NIFTP CD63 positive vesicles appear mainly in the cytoplasm of tumor cells along with EGFR that shows enrichment at the plasma membrane.

High EGFR protein expression was characteristic of PTC, while moderate to low EGFR expression were detected in NIFTP and FND tissues (**Figure 1**; **SI Table 1**). In PTC EGFR protein expression appeared as either granular cytoplasmic or cytoplasmic/membranous, and the two patterns could be simultaneously seen in the same tissue in some cases (**Figure 1**). Enrichment of expression in the basolateral areas of the thyroid follicles was also characteristic of PTC cases (**Figure 1**). In NIFTP and FND EGFR protein expression was mostly cytoplasmic/membranous with moderate to low intensity (**Figure 1**). Double immunofluorescence stain experiments showed colocalization of EGFR with ALIX and CD63 in PTC and NIFTP in intracellular and extracellular vesicles (**Figures 2**, **3**). High expression of ALIX and CD63 was characteristic of PTC tissues compared to NIFTP (**Figures 2**, **3**).

### 3.4 MAPK/ERK and HIF1α pathways activity in thyroid cultured cells

The activity of MAPK/ERK and HIF1α pathways was tested by luciferase assay and found to be downregulated in PTC samples compared to control FND samples (**SI Table 2**). Possible regulation of these pathways by miR-146b-5p and miR-7-5p was also tested. Results showed minimal or no effect of these miRNAs loss or gain of function on the activity of these pathways in cultured cells (**SI Table 2**).

### 3.5 ERK and p38 pathway activity in thyroid tissue samples

**Figure 4:**
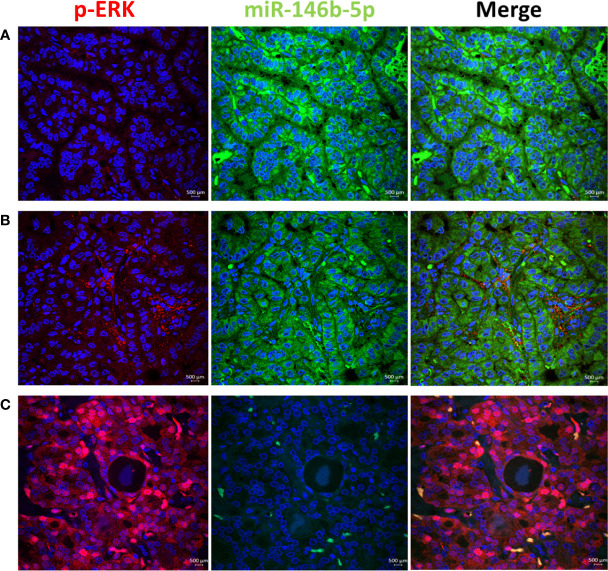
ERK pathway activity in relation to miR-146b-5p expression in representative thyroid tissues. **(A)** In PTC no phospho-ERK (red) is detected in the nuclei of tumor cells with high level of miR-146 (green). **(B)** p-ERK in negative along with low level of miR146b-5p. **(C)** In NIFTP strong nuclear expression of p-ERK is detected in tumor cells with no expression of miR-146b-5p.

**Figure 5:**
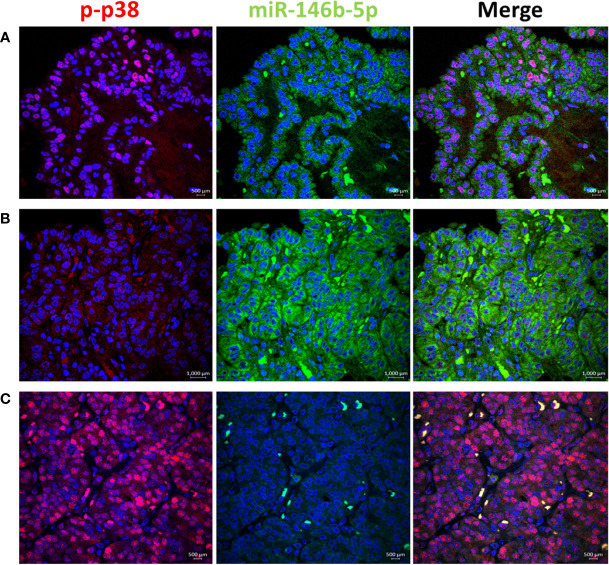
p38 pathway activity in relation to miR-146b-5p expression in representative thyroid tissues. **(A)** In PTC phospho-p38 is detected in the nuclei of tumor cells with low expression of miR-146b-5p. **(B)** p-p38 is negative in areas of miR-146b-5p high expression. **(C)** In NIFTP strong nuclear expression of p-p38 is detected with no expression of miR-146b-5p.

To test the activity of ERK and p38 in real life away from the possible culture related modifying effect, we performed immunofluorescence stain with anti-phospho-ERK1/2 and anti-phospho-p38 antibodies in thyroid tissue FFPE samples. Results showed positive nuclear stain of p-ERK in NIFTP but not in PTC samples (**Figure 4**). All PTC samples, regardless of BRAF mutation status, showed negative p-ERK staining. Positive p-p38 nuclear stain was detected in PTC and NIFTP (**Figure 5**). A heterogenous pattern of negative and positive p-p38 was observed in different samples or different areas of the same tissue in PTC. Real time PCR experiments done on FFPE tissue samples showed that ERK and p38 genes expression are significantly upregulated in NIFTP compared to FND, while in PTC there was no significant change although the proliferation marker KI67 is upregulated (**SI Table 3**). EGF expression was lower in NIFTP and PTC compared to FND (**SI Table 3**).

### 3.6 Factors contributing to reduced EGFR expression in thyroid cultured cells

**Figure 6:**
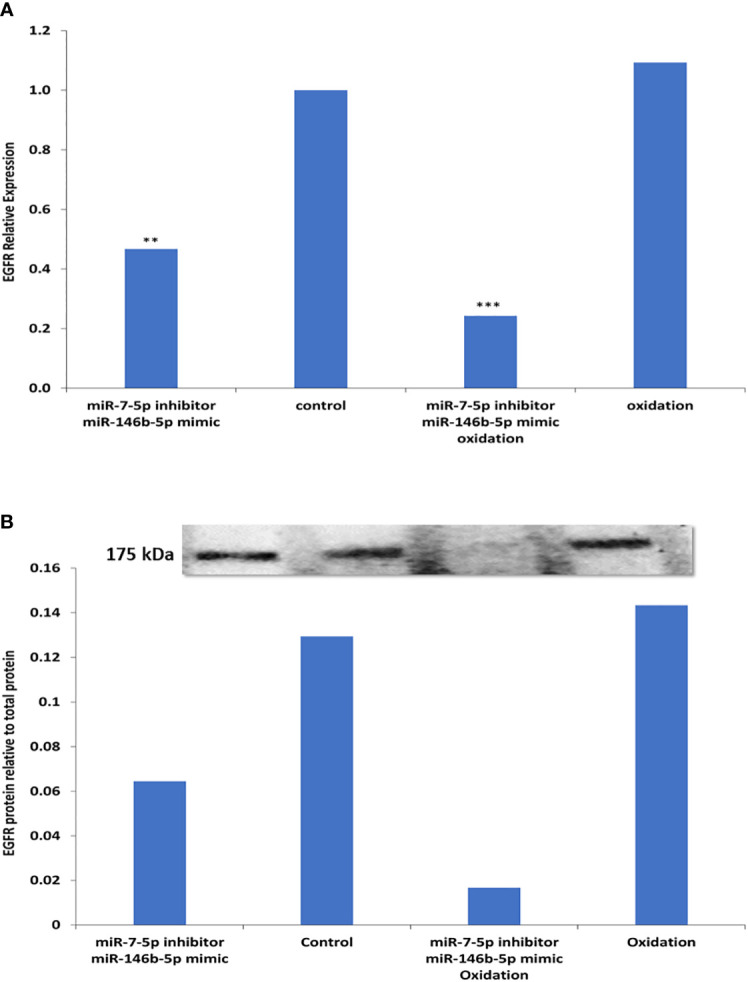
Possible factors contributing to reduced EGFR expression in thyroid cultured cells. **(A)** Expression of EGFR was tested by Real time PCR in five different FND samples. Cells treated with miR-7-5p inhibitor and miR-146b-5p mimic show significant downregulation of EGFR (**p=0.005). Oxidative stress combined with miR-7-5p downregulation and miR-146b-5p upregulation resulted in the highest reduction of EGFR expression (***p < 0.001). **(B)** EGFR protein expression in the same conditions was tested by western blot and showed similar results.

To investigate the possible causes of EGFR downregulation in PTC, we used cultured cells from FND samples and tested the effect of different treatments including miRNAs alteration and oxidative stress that hypothetically simulate the conditions in PTC. Our data shows that the combined effect of miR-7-5p downregulation along with miR-146b-5p upregulation resulted in a significant downregulation of EGFR gene expression (-2.88 folds, p= 0.005, **Figure 6A**). Moreover, oxidative stress combined with miR-7-5p downregulation and miR-146b-5p upregulation resulted in the highest reduction of EGFR expression (-7.25 folds, p= 0.0001, **Figure 6A**). Similar results were detected for EGFR protein expression in the same conditions (**Figure 6B**).

## 4 Discussion

EGFR signaling is a mechanism of autonomous proliferation involved in the pathogenesis of many types of cancer. EGFR binding to its ligand on the cell surface activates signaling pathways, typically the extracellular signal–regulated kinase (ERK) pathway, to promote cell proliferation and survival. The activated receptor is then internalized and transported to the endosomal network where it is either destined for degradation in lysosomes, for termination of signal and maintenance of cellular homeostasis, or recycled to the plasma membrane for prolongation of signal and sustained proliferation (57). An additional fate of internalized EGFR has been described. EGFR is activated by ligand-independent mechanisms, such as stress, and require the activity of the stress activated MAPK-p38 for internalization (57–59). The stress induced EGFR shifts from the canonical trafficking, evade lysosomal degradation and accumulate in endosomes (60–62). The stress-activated receptor can also be recycled to the plasma membrane by p38 inhibition (63). This model of EGFR spatiotemporal control and cell fate has been reported in tumorigenesis and in response to cancer therapy. How the high-risk and low-risk thyroid tumors, specifically PTC and NIFTP, fit into this model?

In NIFTP, our results showed that EGFR mRNA is overexpressed while the EGFR protein expression is low with enrichment at the plasma membrane of tumor cells (**Table 1**; **Figure 1** and **SI Table 1**),. Protein expression is stronger in areas under the capsule compared to the core nodule where a weak cytoplasmic stain is detected indicating loss of the protein (**SI Figure 1**). There is evidence of active signaling through the ERK pathway indicated by nuclear stain with p-ERK (**Figure 4**). The EGF growth factor is expressed although at a lower level than the control FND samples (**SI Table 3**). EGFR internalization is indicated by the cytoplasmic expression of EGFR protein and its co-localization with the endosomes/exosomes markers (ALIX and CD63) (**Figures 2**, **3**). The stress-inducible p38 MAPK pathway is active as indicated by nuclear expression of p-p38 in the tumor cells (**Figure 5**). Altogether, these results suggest that NIFTP growth is dependent on EGFR activity through the canonical pathway where the receptor is activated by ligand binding (EGF or possibly other untested ligands), signals through the ERK pathway, gets internalized in a p38 dependent manner and is subjected to degradation in the cytoplasm.

In PTC, EGFR adopts different pattern of expression and dynamics. Surprisingly in this malignant tumor, EGFR mRNA is downregulated compared to NIFTP and FND (**Table 1**). Moreover, EGFR expression did not correlate with invasion or lymph node metastasis features in our PTC samples (**Table 2**). These results agree with published results which did not find evidence of EGFR increased expression correlating with aggressive features (40). However, these results contradict other studies which could find such correlation (37, 38). We believe that this disagreement is caused by the diagnosis criteria that the authors used at the time of their work which followed the old classification that did not differentiate between PTC and NIFTP. Another surprising result in this study is the lack of ERK activity in all our PTC samples (**Figure 4**). We tested more than 100 PTC tissues by immunofluorescence and found no evidence of active ERK regardless of BRAF mutation presence or absence. Nuclear p-ERK was only seen in stromal cells and normal looking follicles outside the tumor in PTC samples while it was detected in thyroid follicular cells in NIFTP (**Figure 4**). Although PTC is known as a BRAF associated cancer with expected active RAS/ERK pathway (26), search of the literature did not reveal clear pictures of activated ERK in PTC. Our results agree with Lee at al who reported that phospho-ERK1/2 was detected in only eight (4.8%) cases out of 167 PTC samples and the staining intensity or nuclear localization were independent of the BRAFV600E mutation status (64). Moreover, our functional assay in PTC cultured cells showed that the ERK pathway activity is downregulated compared to FND (**SI Table 2**). Therefore, we speculate that inhibition of MAPK/ERK pathway activity might be a mechanism characteristic of malignant cells in PTC. This inhibition can be caused by reduced EGFR tyrosine kinase activity or by some other mechanisms.

At the protein level, our results showed that EGFR is highly expressed in PTC mainly in the cytoplasm and the plasma membrane of tumor cells (**Figure 1**; **SI Table 1**). Landriscina et al. demonstrated that EGFR protein increased expression occur in malignant and less differentiated cells in PTC therefore they concluded that EGFR expression correlate with aggressiveness (39). We found similar increased protein level in malignant tumors compared to benign tumors **Figure 1**; **SI Table 1**). However, this increased protein availability is not due to increased gene expression, considering the low mRNA level in PTC, rather it is the result of accumulation of EGFR protein possibly due to reduced degradation. EGFR accumulation and arrest at non-degradable state in endosomal compartments is usually associated with the stress-induced EGFR internalization. This type of internalization requires activation of the p38 pathway and is independent of the EGFR tyrosine kinase activity (58–62). Our immunofluorescence stain results in PTC samples showed a heterogenous pattern with positive nuclear p-p38 expression in some tissue areas and negative p-p38 in others (**Figure 5**). As activated p38 is required for EGFR internalization and evasion of degradation, inhibition of p38 activity facilitates EGFR recycling to the plasma membrane (63). EGFR expression at the plasma membrane of tumor cells was also observed in our PTC samples which correlate with the p38 heterogenous pattern in our samples. Enrichment of EGFR expression to the basolateral domains of the tumor cells was only observed in our PTC samples with no change in the ERK activation pattern (**Figure 1**). Previous published data showed that EGFR-mediated phosphorylation of certain substrates differ at the apical and basolateral cell membrane and EGFR mis-localization can result in abnormal signaling and aberrant cell behavior (65). Altogether, our results in PTC favor the non-canonical scenario of stress-induced internalization of EGFR with accumulation of non-degraded protein in endosomal compartments and recycling to the plasma membrane in parts of the tumors.

The mechanisms that control the fates of endosomal cargos to degradation, accumulation or secretion is still not clear. It has been shown that intracellular retention of stress-induced EGFR is required for EGFR signaling and protection from apoptosis. This process depends on the endosomal sorting complex required for transport (ESCRT) machinery and ALG-2-interacting Protein X (ALIX) (63, 66). ALIX contributes to many ESCRT-dependent processes such as virus budding, autophagy, and exosome biogenesis (67–69). ALIX was identified as an important regulator of the signaling output of activated EGFR since it mediates its endosomal sorting and rapid silencing; Knockdown of Alix inhibited sorting of the activated EGFR and promoted sustained activation of (ERK)1/2 (70). However, ALIX did not have a role in sorting of EGF-stimulated EGFR or its targeting to lysosomes for degradation (66). Moreover, ALIX was found to mediate the endosomal sorting of tetraspanins, including the exosomal protein CD63, and their secretion *via* exosomes (71, 72). We used ALIX and CD63 as endosomal/exosomal markers to further investigate the fate of EGFR. Our confocal microscopy results showed expression of EGFR in ALIX and CD63 positive intracellular and extracellular vesicles which indicate trafficking of EGFR from late endosomes to exosomal secretion. Increased expression of ALIX was characteristic of PTC samples in this study. It has been shown that overexpression of ALIX reduces the ubiquitylation of EGFR (73) which can explain the increased protein accumulation of EGFR in our PTC samples. Zannetti-Domingues et al. described a role of EGFR in exosome trafficking, biogenesis and uptake by recipient cells, thereby participating in its own dissemination (74). Since exosomes are able to transfer various biological molecules including protein, RNA, DNA, and miRNA, they serve as key modulators of intercellular communication in many physiological and pathological conditions (75, 76). Therefore, multiple roles of exosomal EGFR in metastasis formation and tumor immunity has been described (77–79). This is the first report on EGFR exosomal dissemination in PTC.

Multiple microRNAs have been reported to modulate the expression of EGFR and its downstream signaling pathways in different cancers. It has been reported that miR-7-5p attenuates the activation of ERK signaling and induces cell cycle arrest and cell death in cancer cells by down-regulating EGFR expression (47–51). Our functional assays showed that miR-7-5p and miR-146b-5p regulate the expression of EGFR in PTC cultured cells (**Tables 3** and **4**). Moreover, the combined effect of miR-7-5p downregulation and miR-146b-5p upregulation could reproduce the pattern of low EGFR seen in PTC (**Figure 6**). In PTC tissue samples, miR-146b-5p high expression detected by *in situ* hybridization coexisted with increased EGFR protein expression in PTC tissues (**Figure 1**) which support the idea that EGFR protein presence in PTC is the result of protein accumulation and lack of degradation and not sourced by increased protein translation that would be otherwise inhibited by miR-146b-5p. The effect of miR-146b-5p on ERK activity was not clear in our cultured cells (**SI Table 2**). This can be due to the culturing conditions which possibly change the endogenous ERK activity and mask the regulatory effect of the transfected miRNA. However, in tissue samples there was an inverse association between active ERK and miR-146b-5p expression level (**Figure 4**). Therefore, the regulatory effect of miR-146b-5p on ERK activity cannot be ruled out.

How cancer cells in PTC would benefit from downregulation of EGFR? Although our data is not enough to answer this question, it can direct our attention away from EGFR role as an oncogene towards its role in stress response. It is well established that oxidative stress plays an active role in carcinogenesis and cancer progression (80). High level of oxidative DNA damage was demonstrated in cancer thyroid tissue and proposed to be involved in disease progression through modulation of EGFR and its downstream signaling (81–83). Prolonged EGFR signaling and accumulation of stress- activated receptor creates a feedback mechanism which leads to apoptosis (84, 85). In our previous work we showed that miR-146b-5p has an inhibitory effect on the stress MAPK/JNK/AP1 pathway in PTC and protects thyroid cells from cell death in response to oxidative stress (55). Previous published results showed that EGFR contributes to the cellular response to stress by upregulating the transcription of the EGFR gene and modulating miRNA biogenesis in tumor cells (86, 87). Our results here show that in the presence of high level of miR146b-5p, EGFR is downregulated in response to oxidative stress (**Figure 6**). Altogether these results suggest that EGFR upregulation can be part of the stress response in thyroid cells, and miR-146b-5p increased expression in PTC downregulates EGFR possibly to protect the cells against stress-induced cell death.

## 5 Conclusion

**Figure 7:**
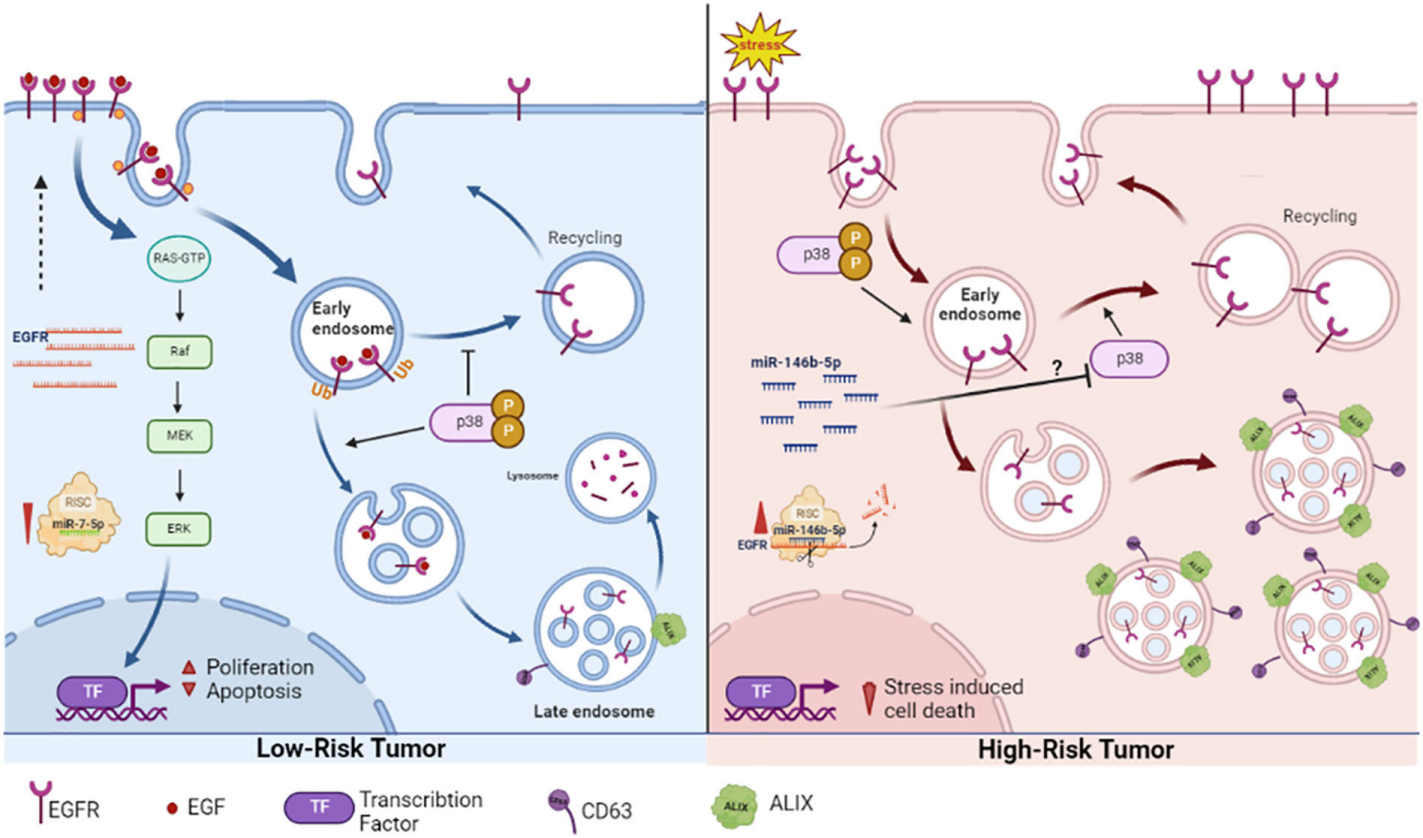
Our proposed EGFR expression regulation and spatial dynamics in low risk (NIFTP) and malignant (PTC) thyroid neoplasms. In NIFTP, EGFR transcripts are abundant in association with reduced level of miR-7-5p. EGFR protein at the plasma membrane binds to its ligand, activates the ERK pathway to stimulate proliferation. The activated receptor is then internalized into the endosomal network where it is destined for degradation. Active p38 favors internalization and represses recycling of EGFR to the plasma membrane. In PTC, EGFR transcript level is low in association with high level of miR-146b-5p and high stress levels. The stress activated EGFR gets internalized, evades lysosomal degradation and accumulates in endosomes/exosomes. The ERK pathway is not active. Inhibition of p38 possibly by miR-146b-5p supports recycling of EGFR to the plasma membrane. Created using BioRender.com.

This work provides a new perspective of the already known increase EGFR protein associated with malignancy. High EGFR protein in PTC (high risk tumor) is not due to gene overexpression, but rather caused by accumulation of non-degraded protein arrested in endosomal compartments and disseminated through exosomes to the extracellular milieu. In PTC EGFR does not signal through ERK pathway and may be involved in the stress response during carcinogenesis. In NIFTP (low-risk tumor) EGFR is overexpressed and follows the canonical pathway of signaling through ERK pathway, internalization and recycling to plasma membrane and ending with degradation [**Figure 7**]. These patterns can be used as diagnostic features in addition to the histopathological characterization. The different regulation patterns differentiating the high risk from low-risk tumors in this work may open new windows for investigating and targeting the intracellular trafficking associated with malignancy. This work also showed two microRNAs that have combined effect on EGFR which may have important implications in EGFR related cancers prognosis and therapy.

## Data availability statement

The original contributions presented in the study are included in the article/supplementary material. Further inquiries can be directed to the corresponding author.

## Ethics statement

The studies involving human participants were reviewed and approved by Kuwait ministry of health. The patients/participants provided their written informed consent to participate in this study.

## Author contributions

AA-A conceived the idea, planned the experiments, analyzed the data and wrote the manuscript. IJ performed the experiments, helped in data analysis and prepared tables and figures. RA and NA-B collected the samples, did histopathological classification and immunohistochemistry scoring. JP did the tissues immunostaining. BA-S helped in cell culture and functional assays. MA-B helped in data analysis. All authors contributed to the article and approved the submitted version.

## Supplementary material

Supplementary Figure 1 NIFTP case showing protein expression that is stronger in areas under the capsule compared to the core nodule where a weak cytoplasmic stain is detected indicating loss of the protein. Intensity of EGFR expression negatively correlates with expression of miR-146b-5p detected by *in situ* hybridization (purple color).
