# Supplementary figures and images for: A new paradigm for epidermal growth factor receptor expression exists in PTC and NIFTP regulated by microRNAs
Source: Front Oncol. 2023 Apr 11;13:1080008. doi: 10.3389/fonc.2023.1080008 (PMC10126268; doi:10.3389/fonc.2023.1080008)

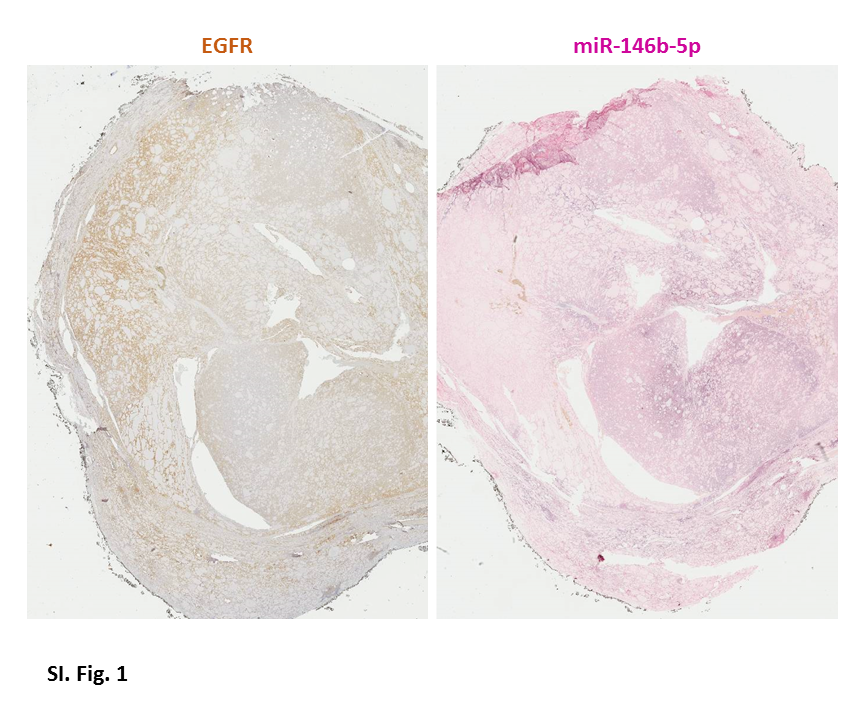

Supplement: Supplementary Figure 1 — NIFTP case showing protein expression that is stronger in areas under the capsule compared to the core nodule where a weak cytoplasmic stain is detected indicating loss of the protein. Intensity of EGFR expression negatively correlates with expression of miR-146b-5p detected by in situ hybridization (purple color). [file Image_1.tif]
