# Supplementary material for: A new paradigm for epidermal growth factor receptor expression exists in PTC and NIFTP regulated by microRNAs
Source: Front Oncol. 2023 Apr 11;13:1080008. doi: 10.3389/fonc.2023.1080008 (PMC10126268; doi:10.3389/fonc.2023.1080008)
Supplement: Supplementary file 2 [file Table_1.docx]

**SI Table 1: EGFR protein expression in thyroid tissues by immunohistochemistry**

| **Diagnosis** | **Low EGFR** | **High EGFR** | **Subcellular localization** | **p-Value*** |
| --- | --- | --- | --- | --- |
| **cPTC (n=25)** | 0 | 25 | Cytoplasmic granular or Cytoplasmic/Membranous | < 0.00001 |
| **NIFTP (n=17)** | 10 | 7 | Cytoplasmic/Membranous | 0.066 |
| **FND (n=10)** | 10 | 0 | Membranous | - |

Low EGFR: 1+ intensity in any percentage or 2+ intensity in <50% of tumor cells. High EGFR: 2+ staining of ≥50% of tumor cells or 3+ staining of any percentage of tumor cells. * Fisher's exact tests were used to compare the groups.
