# Supplementary material for: A new paradigm for epidermal growth factor receptor expression exists in PTC and NIFTP regulated by microRNAs
Source: Front Oncol. 2023 Apr 11;13:1080008. doi: 10.3389/fonc.2023.1080008 (PMC10126268; doi:10.3389/fonc.2023.1080008)
Supplement: Supplementary file 3 [file Table_2.docx]

| **PTC samples** | **Endogenous miRNA expression level^a^** | | **Endogenous Signaling Pathway activity^b^** | | **miRNA expression level post transfection** | | | **Signaling Pathway activity post transfection^c^** | |
| --- | --- | --- | --- | --- | --- | --- | --- | --- | --- |
|  |  | | Hypoxia | MAPK/ERK |  | | | Hypoxia | MAPK/ERK |
|  | miR-146b-5p level | miR-7-5p level | HIF1α | Elk-1/SRF | miR-146b-5p level | | miR-7-5p level | HIF1α | Elk-1/SRF |
| **1** | 10.76 | -22.85 | -7.15 | -7.39 | -7.82 | - | | -1.57 | -1.02 |
| **2** | 15.99 | -15.37 | -13.02 | -2.09 | -47.72 | - | | 1.50 | -1.00 |
| **3** | 2.12 | -13.53 | -1.74 | -1.93 | -5.85 | - | | -3.21 | -1.26 |
| **4** | 3.78 | -6.80 | 2.90 | 3.23 | -1.27 | - | | -2.94 | -1.61 |
| **5** | 29.05 | -31.71 | -2.77 | -2.18 | -43.07 | - | | -1.06 | -1.32 |
| **6** | 1.60 | -12.14 | -3.05 | -3.94 | -1.63 | - | | 1.35 | 1.46 |
| **7** | 1.09 | -17.72 | -6.56 | -7.80 | -50.00 | - | | -1.46 | -1.33 |
| **8** | 14.68 | -2.38 | -5.67 | -6.45 | - | 18.65 | | 3.58 | 1.49 |
| **9** | 5.24 | -62.77 | -7.60 | -2.77 | - | 4.03 | | 2.98 | 1.11 |
| **10** | 3.01 | -79.73 | -1.75 | -1.70 | - | 19.31 | | 7.37 | 8.54 |
| **11** | 7.80 | -43.74 | -5.38 | -2.75 | - | 4.46 | | 2.33 | 1.04 |
| **12** | 6.23 | -17.40 | -2.84 | -1.50 | - | 2.39 | | -1.97 | -1.21 |

**SI Table 2: Signaling pathways activity in PTC in relation to miR-146b-5p and miR-7-5p expression level changes**

a: miRNA level expressed in fold change compared to FND; b: Pathways activity expressed as fold change of the luciferase activity of PTC cells compared to FND. c: Pathways activity expressed as fold change of the luciferase activity of cells transfected with miRNA inhibitor or mimic compared to those transfected with negative control.
