# Supplementary material for: A new paradigm for epidermal growth factor receptor expression exists in PTC and NIFTP regulated by microRNAs
Source: Front Oncol. 2023 Apr 11;13:1080008. doi: 10.3389/fonc.2023.1080008 (PMC10126268; doi:10.3389/fonc.2023.1080008)
Supplement: Supplementary file 4 [file Table_3.docx]

|  | **∆Ct** | | | **Fold Change** | | **p-value*** | |
| --- | --- | --- | --- | --- | --- | --- | --- |
|  | **CPTC (n=30)** | **NIFTP (n=14)** | **FND (n=8)** | **CPTC** | **NIFTP** | **CPTC** | **NIFTP** |
| **EGF** | 6.22 | 5.87 | 3.15 | -8.40 | -6.56 | 0.067 | 0.059 |
| **ERK** | 1.48 | 0.02 | 1.62 | 1.10 | 3.03 | 0.688 | 0.040 |
| **P38** | 0.29 | -2.26 | 2.02 | 3.31 | 19.46 | 0.103 | 0.006 |
| **MKI67** | 1.74 | 1.84 | 4.79 | 8.28 | 7.71 | 0.058 | 0.022 |

**SI Table 3: Expression of genes related to EGFR pathway**

Expression is calculated using the relative quantification method (ΔCt = CT of target normalized to CT of housekeeping gene). Fold change is calculated using (2^–ΔΔCT^) formula. *Statistical analysis is done using student *t*-test.
